# Spatial odor discrimination in the hawkmoth, *Manduca sexta* (L.)

**DOI:** 10.1242/bio.058649

**Published:** 2021-03-26

**Authors:** Kalyanasundaram Parthasarathy, M. A. Willis

**Affiliations:** Department of Biology, Case Western Reserve University, Cleveland, OH 44106-7080, U.S.A

**Keywords:** Olfaction, Odor localization

## Abstract

Flying insects track turbulent odor plumes to find mates, food and egg-laying sites. To maintain contact with the plume, insects are thought to adapt their flight control according to the distribution of odor in the plume using the timing of odor onsets and intervals between odor encounters. Although timing cues are important, few studies have addressed whether insects are capable of deriving spatial information about odor distribution from bilateral comparisons between their antennae in flight. The proboscis extension reflex (PER) associative learning protocol, originally developed to study odor learning in honeybees, was used as a tool to ask if hawkmoths, *Manduca sexta,* can discriminate between odor stimuli arriving on either antenna. We show moths discriminated the odor arrival side with an accuracy of >70%. Information about spatial distribution of odor stimuli may be available to moths searching for odor sources, opening the possibility that they use both spatial and temporal odor information.

This article has an associated First Person interview with the first author of the paper.

## Abstract (precis)

**Summary:** The proboscis extension reflex (PER) associative learning protocol was used as a tool to ask if hawkmoths, *Manduca sexta*, can discriminate between odor stimuli arriving on either antenna.

## INTRODUCTION

Animals with bilaterally symmetrical sensory structures (i.e. eyes, ears, nostrils, antennae, etc.) assess information by either combining and comparing bilateral inputs from sequential time points (temporal sampling), or comparing bilateral inputs at the same time point (spatial sampling) (Dusenbery, 1992; Fraenkel and Gunn, 1961; Schöne, 1984). Several studies have shown that a variety of animals use bilaterally sampled inputs from their olfactory organs to orient towards odor sources (Ando and Kanzaki, 2015; Borst and Heisenberg, 1982; Catania, 2013; Duistermars et al., 2009; Gardiner and Atema, 2010; Khan et al., 2012; Kraus-Epley and Moore, 2002; Louis et al., 2008; Martin, 1965; Page et al., 2011; Parthasarathy and Bhalla, 2013; Porter et al., 2007; Rajan et al., 2006; Takasaki et al., 2012; Vonbekesy, 1964; Wasserman et al., 2012; Weissburg and Dusenbery, 2002). These studies employ a variety of techniques including genetic manipulations in controlled laboratory environments (Gomez-Marin et al., 2011; Louis et al., 2008), unilateral odor stimulation in tethered walking and flying preparations (Borst and Heisenberg, 1982; Duistermars et al., 2009; Takasaki et al., 2012), telemetric unilateral stimulation of freely swimming sharks (Gardiner and Atema, 2010), and experimentally manipulated individuals in naturalistic environments (Catania, 2013; Gardiner and Atema, 2010; Khan et al., 2012; Kraus-Epley and Moore, 2002; Parthasarathy and Bhalla, 2013; Rajan et al., 2006; Takasaki et al., 2012). Despite a separation between sensory organs ranging from less than a millimeter (Louis et al., 2008) to multiple centimeters (Gardiner and Atema, 2010), asymmetric stimulation usually caused asymmetric steering or the inability to maintain orientation to stimulus (Gardiner and Atema, 2010; Louis et al., 2008). Odor asymmetry was detected when presented as a bilateral difference in odor concentration or onset timing (Gardiner and Atema, 2010; Parthasarathy and Bhalla, 2013; Rajan et al., 2006; Vonbekesy, 1964).

Previous studies assumed that bilaterally located sensors enhance spatial sampling. However, studies on plume tracking during flight typically assume that insects do not spatially sample odors, because turbulent odor plumes may not provide predictable directional cues leading to the source. Also, freely flying plume trackers may move too fast for their nervous systems to process bilateral asymmetries and maneuver accordingly. Directional cues are thought to be provided by the wind or water flow detected visually (Emanuel and Dodson, 1979; Fry et al., 2009; Kennedy, 1940; Kennedy and Marsh, 1974), or via mechanosensation (Baker and Montgomery, 1999; Kulpa et al., 2015). Turning maneuvers underlying zigzagging trajectories are thought to be pre-programmed in the central nervous system and expressed in response to an attractive odor (Kennedy, 1983; Arbas et al., 1993; Mafra-Neto and Cardé, 1994; Vickers and Baker, 1994). Bilateral sampling has been deemed unnecessary (Arbas et al., 1993).

Few studies have directly addressed if bilateral olfactory comparisons contribute to plume tracking in moths (Ando and Kanzaki, 2015; Takasaki et al., 2012; Vickers and Baker, 1991). Experiments in which male *Heliothis virescens* (Fabricius) moths tracked a pheromone plume with one antenna removed showed minor changes in their flight trajectories and interestingly, removal of an antenna diminished successful plume tracking by roughly ∼50% relative to the intact controls (Vickers and Baker, 1991). This result was attributed to the overall loss of odor inputs and timing of odor onset resulting from moths with asymmetric antennae encountering the edges versus the centerline of a laboratory-generated odor plume. The moth's inability to make bilateral comparisons across two antennae was discounted (Vickers and Baker, 1991). In contrast, Takasaki et al. showed that walking silk moths, *Bombyx mori* (L.) alter their steering in response to asymmetric odor stimulation (Ando and Kanzaki, 2015; Takasaki et al., 2012). However, they cautioned that walking and flying are very different modes of locomotion. Because flying insects move much faster than walking insects, they may use bilateral odor information differently (Takasaki et al., 2012). Indeed, the use of spatial sampling during plume tracking in flight remains an open question, even though odor plume tracking is a well-studied topic in flying moths and flies tracking attractive odors (Willis and Arbas, 1991; Cardé and Willis, 2008; Duistermars et al., 2009; Saxena et al., 2018; van Breugel and Dickinson, 2014).

Before we try to understand the role of spatial sampling in plume tracking during flight, we must first establish that moths are capable of discriminating between olfactory stimuli detected at one antenna versus the other. We studied this question in the hawkmoth *M. sexta* using an associative learning paradigm based on the proboscis extension reflex (PER) conditioning developed for honeybees (Kuwabara, 1957). Building on previous studies that used PER to demonstrate associative learning in hawkmoths (Daly and Smith, 2000; Daly et al., 2001, 2008), we developed a behavior paradigm to probe the spatial odor representation in moths. In our study, trained moths discriminated the odor arrival side with an accuracy of >70%. Thus, moths can distinguish the odor arrival side and may be capable of extracting spatial information about the odor distribution while tracking plumes in flight.

Studies on sensory control of behavior routinely deliver experimentally controlled stimuli and measure the organism's response (i.e. cell, circuit, or behavior) relative to stimulus onset, offset and/or duration. Most animals studied are bilaterally symmetric, with paired symmetrical sensory structures, but the impact of their asymmetrical activation is often ignored. In this study, we tested the ability of *M. sexta* to sense odor-arrival direction by stimulating asymmetrically. We show that moths can discriminate odor arrival on one antenna relative to the other using an associative learning test.

## RESULTS AND DISCUSSION

Using an associative learning protocol, we asked female *M. sexta* moths if they could treat odor input to each antenna independently. Their behavioral response showed that most individuals learned to associate odorant-arrival side with sucrose reward. In our experimental population, the number of moths that learned the linalool and benzaldehyde arrival side with reward were 68% (19/28) and 85% (17/20), respectively (Fig. 2A and D). We observed that more experimental moths learned to associate benzaldehyde with a sugar reward compared to the odorant linalool. Linalool is a common plant odor that is known to induce oviposition behavior in mated female moths and is also a component of floral scent (Bisch-Knaden et al., 2018; Raguso, 2016; Reisenman et al., 2010; Saveer et al., 2012). On the other hand, benzaldehyde could be categorized as a floral scent, as it is emitted by many species of night blooming flowers and is known to act as a pollinator attractant (Raguso et al., 2003a,b). It may be that *M. sexta* females are more likely to associate the ecologically relevant conditioning stimulus of a sucrose meal with a known floral scent like benzaldehyde, than the known oviposition stimulus linalool. Further studies are required to resolve that question.

When tested, moths activated their feeding response in >70% of the trials (left: 74±5.6%; right: 78±9.65%) when linalool arrived on the antenna associated with the reward. The same moths performed poorly (left: 21±8%; right: 24±8.9%) when linalool was delivered to the unassociated side (Fig. 2B; Left *n*=9; Fig. 2C; Right *n*=10). Generalized linear mixed-effects model (GLMM) analysis (see Materials and Methods) showed a strong effect of odor arrival side on moth's response, with negligible variability between individuals (Table 1). The estimated electromyogram (EMG) response probabilities were higher (0.75 and 0.74) when linalool was presented on the same side as the associated side, and lower (0.27 and 0.28) when presented on the unassociated side (see Table S2).

**Table 1.**
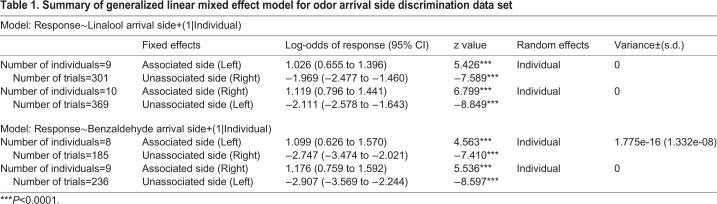
**Summary of generalized linear mixed effect model for odor arrival side discrimination data set**

We observed a similar pattern, when we tested moths that associated benzaldehyde arrival on a specific antenna with reward. When moths experienced benzaldehyde on the associated side, they showed an EMG response for more than 70% of stimulus presentations (left: 75±5.4%; right: 78±7.2%). While the same odor was given to unassociated side, the moths response was poor as they responded only to 16±6.6% of left and 15±6.8% right side stimulus presentations (Fig. 2E, left *n*=8; Fig. 2F, Right *n*=9). Using GLMM analysis, we again determined the effects of benzaldehyde arrival side on moth's response. The odorant arrival side strongly contributed to moth's response (Table 1) and the estimated probabilities (see Table S3) were high (0.70 and 0.76) for associated side and low for unassociated side (0.16 and 0.15). GLMM showed that performance variability between individuals in odor-arrival side discrimination task was negligible (Table 1).

Animals with only one of their normally bilaterally symmetric odor sensors (i.e. antennae in insects and nostrils in mammals) have been shown to take more time to locate an odor source and their probability of locating the source is reduced (Vickers and Baker, 1991; Sanders and Lucuik, 1992; Lockey and Willis, 2015; Rajan et al., 2006; Porter et al., 2007; Khan et al., 2012; Catania, 2013; Kraus-Epley and Moore, 2002). The loss of one of the sensors may not only reduce the received odor input it is also likely to remove the spatial representation of odor information.

The ability to extract odor direction information during plume tracking could prove to be useful especially when the cross-section of the odor plume becomes narrow near the source. In addition, moths typically slow down and transition to hovering flight as they approach the odor source (Willis and Baker, 1994; Willis and Arbas, 1998). Near the source, even small lateral movements of the moths’ flight trajectory may introduce a bias in the amount of odor experienced by each antenna. Since *M. sexta's* antennal tips are held around 2 cm apart in flight, near the source one of the antennae is likely to be either fully or partially outside the plume. This asymmetrical odor experience would not only inform the moth about loss of odor in one the antennae but also about its position relative to the plume boundary. When foraging in a floral patch, the moth's choice of flower to feed from may depend on the spatial odor information i.e., right or left, along with visual cues. This directional information could be extracted by comparing the odor inputs received across the antennae.

Such a bilateral comparison would require the laterality of the odor information to be preserved until it reaches higher brain regions where bilateral information is processed. Neural recordings from the lateral accessary lobe (LAL) region of the hawkmoth brain showed neurons responding to ipsilateral odor stimulation, suggesting that laterality of the odor information is maintained in higher brain regions (Kanzaki et al., 1991). Additionally, both left and right LALs are connected via a commissure of lateral accessory lobe and bilateral odor information is thought to be integrated in the LALs (Homberg et al., 1988; Kanzaki et al., 1991).

Taken together, these findings suggest that *M. sexta* moths likely increase their use of bilaterally sampled odor distribution in the near-source plume to locate the odor source. We demonstrate here that *M. sexta* can discriminate between asymmetric odorant onsets between its antennae, thereby opening several questions about odor modulation of plume tracking that may have previously escaped our notice.

## MATERIALS AND METHODS

### Animals

We used 4-day-old virgin female *M. sexta* (L.) moths reared in our laboratory and maintained on a 14:10 light:dark cycle. Experiments were conducted during the dark phase of the L:D cycle when plume tracking typically occurs in nature (Sasaki and Riddiford, 1984). Moths had access to sucrose solution only during the experiments.

### Experimental setup

#### Odor delivery system

**Fig. 1.**
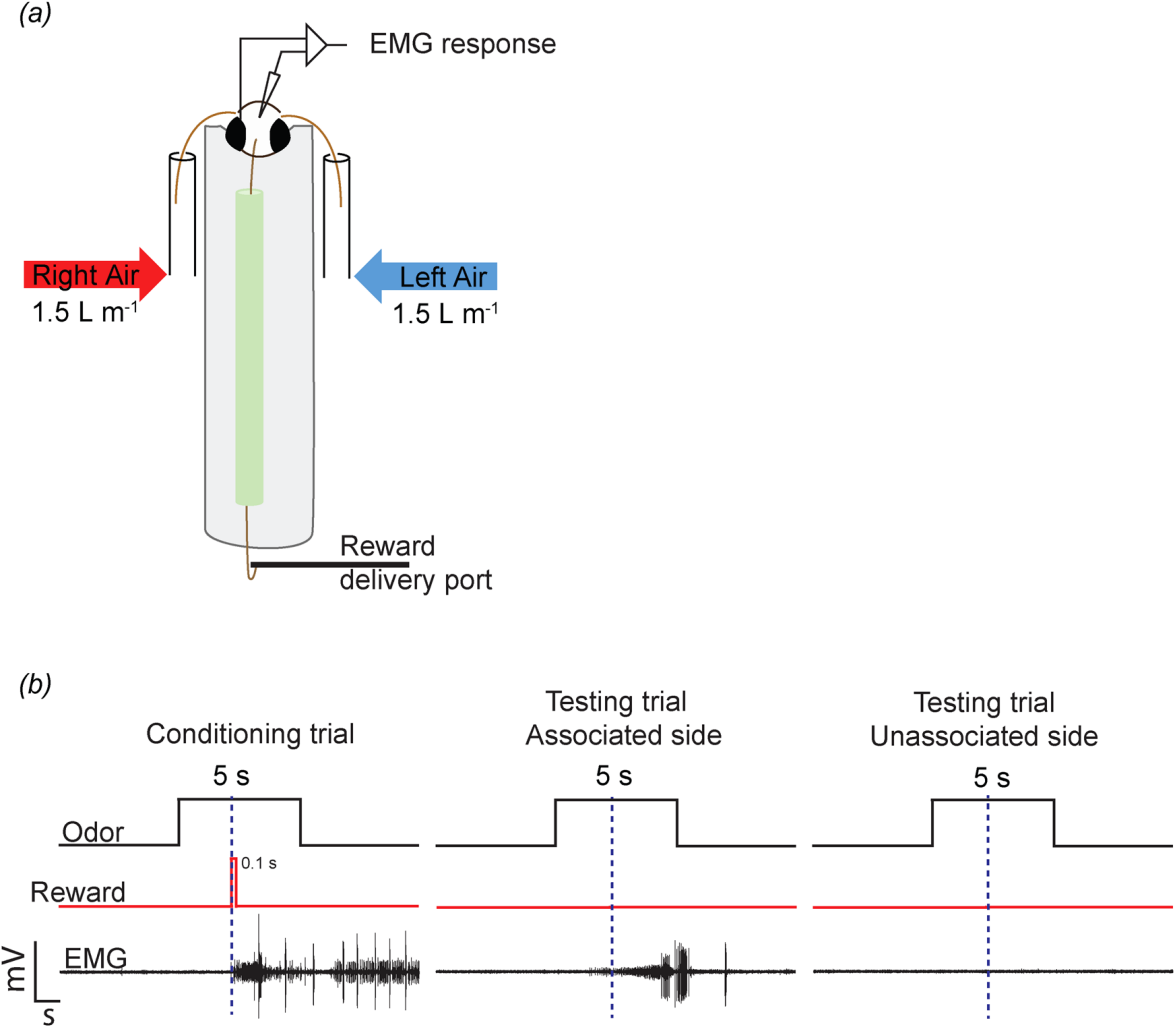
**Behavioral setup and training protocol.** (A) Schematic diagram of behavioral setup showing a restrained moth with its straightened proboscis and odor delivery onto its antennae. Reward delivery port was placed at the distal tip of the proboscis. The feeding response was monitored using EMG activity of the suction pump muscles in the head. (B) Schematic diagram shows events during training and test protocols. Odor was presented for 5 s and 3 s after odor onset the reward valve was activated for 0.1 s. Moths were not rewarded during test session.

Each antenna (left or right) received odor from a custom-built odor delivery system consisting of blank, empty glass vials and a glass vial containing a Whatman number 1 filter paper strip (0.5×10 mm) soaked with neat odorant. The stimulus was produced by allowing clean air (1.5 L/min) to flow through the glass vial containing saturated odor vapor, and odorized air was injected into the tube delivering odor to an antenna (Fig. 1A). Air was filtered using a charcoal filter and bubbled through water to humidify it before reaching the odor vials. Odor vials were changed every day and fresh odor loaded into the vial before every training session. During inter-trial intervals, air flowed through the blank vial to ensure constant flow of air onto the moth. Each antenna was inserted into separate independent odor delivery tube (1.6 mm id, 3.2 mm od; ThermoFisher Scientific, USA) to eliminate cross-contamination between antennae (Fig. 1A). An exhaust fan placed directly above the preparation, removed stimuli away from the room. Airflow in the olfactometer was gated by solenoid valves (Clippard Instrument Laboratory, Fairfield, OH, USA; ET-2M-12H) interfaced with a computer using a two-channel relay controller (National Control Devices, Osceola, MI, USA; R210RS), driven by a custom MATLAB code. During a trial, odorized air was delivered to one antenna while the other received flow-speed-matched clean air from an empty blank vial.

**Fig. 2.**
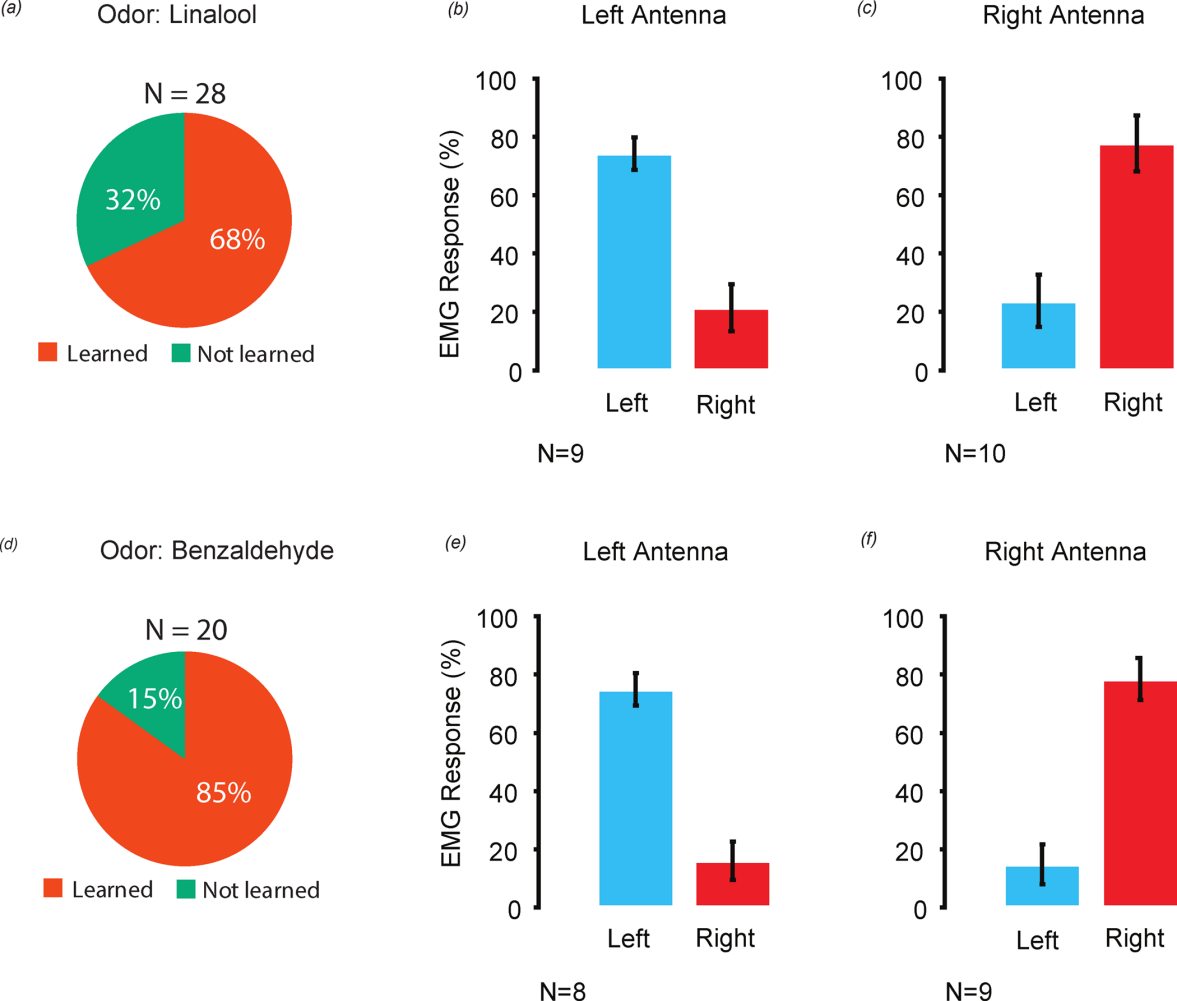
**Odor-arrival side discrimination response.** Pie charts show the number of moths that learned the odor- arrival side task for both linalool (A) and benzaldehyde (D). Bar graphs showing EMG % response for both left (blue bar) and right (red bar) side odor delivery. Panels B and E show the moths’ response when their left antenna was conditioned, and panels C and F when their right antenna was conditioned. (B,C) Moths discriminated linalool arrival side. Response to the associated side was higher, >70% compared to the unassociated side, <30%. (C) Moths discriminated benzaldehyde arrival side. Response to the associated side was higher, >70% compared to the unassociated side, <25%. (B,C,E and F) Error bars indicate standard error of mean.

We used the odorants linalool and benzaldehyde (purity ≥99%, Sigma-Aldrich, USA) to serve as conditioned stimuli. Based on available literature on odor coding in antennal lobes, these two chemicals activate different elements of odor detection and processing circuitry (Bisch-Knaden et al., 2018; Martin et al., 2011; Saveer et al., 2012). We assumed that any observed associations would be general responses. These two compounds have been identified in scents released by night-blooming flowers on which *M. sexta* feed (Raguso et al., 2003a,b). They also attract and stimulate feeding in *M. sexta* (Reisenman et al., 2010; Riffell et al., 2009).

### Training protocol

An intact moth was held immobile in a 6.4 cm long piece of 10 cm diameter copper tubing, with the head attached to the tube surface using soft dental wax (Fig. 1A). Reward (10% sucrose in water) was delivered to the tip of the moths’ experimentally extended 7.5 cm long proboscis (i.e. proboscis was threaded through a 4 cm long, 0.4 cm diameter plastic tube). The tube was cut lengthwise to allow rapid placement of the proboscis. The distal tip of the proboscis was free to coil around the reward delivery tube. A pinch valve (161P010; West Caldwell, NJ, USA) was used to control the reward delivery duration. We monitored feeding attempts by recording electromyograms (EMGs) through a pair of Teflon-coated silver wires (bare diameter 0.127 mm; A-M systems, Sequim, WA, USA) (Daly and Smith, 2000) implanted in the cibarial pump muscle, which when activated draws nectar along the proboscis. Use of cibarial muscle EMGs to study associations in this preparation was developed previously (Daly and Smith, 2000) because tethered *M. sexta* moths do not extend their proboscis like honeybees. EMG signal was filtered 0.1 Hz to 1 KHz and amplified 100 times using a differential amplifier (Warner Instruments, Hamden, CT, USA). We scored each trial using amplified EMG signal played through a loud speaker. Pilot data suggested that our moths required roughly ten trials to associate odor arrival side with a sucrose reward. Initially, moths were given ten to twelve trials of conditioning stimuli i.e., odor was presented for 5 s to one of the antennae, and 3 s after odor stimulus onset, they were rewarded with 3 µl of 10% sucrose solution. After training, moths were tested with trials in which odor arrived either on the associated or the unassociated side according to a pseudo-random list. Moths were expected to generate feeding muscle EMGs when odor was presented to the antenna on the associated side, and no muscle activity during odor presentation to the opposite antenna (Fig. 1B). Each trial was followed by an inter-trial interval ranging 30–60 s randomly decided by the control software. In order to increase the number of test trials performed by moths a few ‘associated side’ trials were randomly rewarded with varying probability (0.18–0.40). Test trials were continued until the moths stopped responding to the stimuli. This was variable, ranging from 23 to 60 trials. Trial details of each individual moth are provided in the supplementary document (Table S1).

### Data analysis

#### Response quantification

Response of the moth was scored based on presence (attempted feeding) or absence (no response) of EMG activity. We sorted the test trials based on odor arrival side and for each odor arrival side, we computed the moth's performance using the following formula:
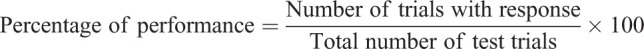


#### GLMM

We analyzed the effect of odor arrival side on the observed behavioral response, i.e., the presence or absence of EMG response by individual moths during the trials and estimated the response variability across individuals using GLMM with binomial distribution. We used lme4 package in the statistical software R to analyze the data (Bates et al., 2015; R Core Team, 2019). The GLMM used is as follows:
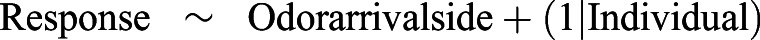
In the model, odor arrival side specified the fixed effect and random effects was specified by (1|Individual).

## Supplementary Material

Supplementary information
