# Supplementary material for: Spatial odor discrimination in the hawkmoth, Manduca sexta (L.)
Source: Biol Open. 2021 Mar 26;10(3):bio058649. doi: 10.1242/bio.058649 (PMC8015231; doi:10.1242/bio.058649)
Supplement: Supplementary information [file biolopen-10-058649-s1.pdf]

Supplementary information:

Table S1. Trial details of each individual moth.

S. No	Moth ID	Odor	Association Side	Total number of training trials	Test trials		
					Associated side	Unassociated side	Rewarded associated side
1	20160317M2	Linalool	Right	11	29	19	11
2	20160603M6	Linalool	Right	12	29	22	8
3	20160607M4	Linalool	Right	12	14	13	4
4	20160610M4	Linalool	Right	12	25	25	9
5	20160615M3	Linalool	Right	12	23	19	7
6	20160616M2	Linalool	Right	12	25	25	9
7	20160621M2	Linalool	Right	12	13	11	3
8	20160622M1	Linalool	Right	12	14	12	3
9	20160624M1	Linalool	Right	12	11	10	2
10	20160628M2	Linalool	Right	12	16	14	4
11	20160602M3	Linalool	Left	11	13	15	5
12	20160603M4	Linalool	Left	12	25	25	8
13	20160606M3	Linalool	Left	11	25	25	10
14	20160607M3	Linalool	Left	12	12	16	4
15	20160609M1	Linalool	Left	12	18	22	7
16	20160613M3	Linalool	Left	12	10	12	3
17	20160623M1	Linalool	Left	12	16	13	4
18	20160627M2	Linalool	Left	12	15	12	5
19	20160627M4	Linalool	Left	12	10	17	4
20	20160714M1	Benzaldehyde	Right	12	19	17	6
21	20160726M2	Benzaldehyde	Right	12	14	12	3
22	20160727M2	Benzaldehyde	Right	12	14	12	3
23	20160729M2	Benzaldehyde	Right	12	16	13	4
24	20160802M1	Benzaldehyde	Right	12	14	12	3
25	20160803M2	Benzaldehyde	Right	12	13	11	3
26	20160804M1	Benzaldehyde	Right	12	13	11	3
27	20160809M2	Benzaldehyde	Right	12	10	12	3
28	20160811M2	Benzaldehyde	Right	12	10	13	3
29	20160726M3	Benzaldehyde	left	12	10	13	3
30	20160726M1	Benzaldehyde	Left	12	13	11	3
31	20160728M2	Benzaldehyde	Left	12	10	14	3
32	20160801M3	Benzaldehyde	Left	12	12	10	3

Biology Open • Supplementary information

33	20160802M3	Benzaldehyde	Left	12	10	14	3
34	20160803M1	Benzaldehyde	Left	12	13	11	3
35	20160809M1	Benzaldehyde	Left	12	12	10	3
36	20160811M1	Benzaldehyde	Left	12	12	10	3

Table S2. Estimated probabilities of response for linalool arrival side, with confidence intervals, obtained by GLMM.

Model: *Response ~ Linalool arrival side + (1|Individual)*		
	**Fixed effects**	**Estimated probability (95% CI)**
Number of individuals = 9<br>Number of trials = 301	Associated side (Left)	0.75 (0.69 to 0.81)
	Unassociated side (Right)	0.27 (0.19 to 0.37)
Number of individuals = 10<br>Number of trials = 369	Associated side (Right)	0.74 (0.69 to 0.80)
	Unassociated side (Left)	0.28 (0.19 to 0.39)

Table S3. Estimated probabilities of response for Benzaldehyde arrival side, with confidence intervals, obtained by GLMM.

Model: *Response ~ Benzaldehyde arrival side + (1|Individual)*		
	**Fixed effects**	**Estimated probability (95% CI)**
Number of individuals = 8<br>Number of trials = 185	Associated side (Left)	0.75 (0.65 to 0.83)
	Unassociated side (Right)	0.16 (0.9 to 0.28)
Number of individuals = 9<br>Number of trials = 236	Associated side (Right)	0.76 (0.68 to 0.83)
	Unassociated side (Left)	0.15 (0.8 to 0.26)

Biology Open • Supplementary information
